# Mental health, risk behaviors, and social life factors in relation to adolescents’ suicide ideation, plans and attempt

**DOI:** 10.1007/s00787-024-02616-2

**Published:** 2024-11-15

**Authors:** Stine Danielsen, Katrine Strandberg-Larsen, Massimiliano Orri, Merete Nordentoft, Annette Erlangsen, Trine Madsen

**Affiliations:** 1 Danish Research Institute for Suicide Prevention– DRISP, Mental Health Center Copenhagen, Gentofte Hospitalsvej 15, Opg. 15, 4. floor, DK– 2900 Hellerup, Denmark; 2 Section of Epidemiology, Department of Public Health, Faculty of Health and Medical Sciences, University of Copenhagen, Øster Farimagsgade 5, bd. 24, PO Box 2099, Copenhagen, DK - 1014 Denmark; 3 McGill Group for Suicide Studies, Department of Psychiatry, Douglas Mental Health University Institute, McGill University, 6875 Bd LaSalle, H4H 1R2 Montreal, Verdun, Quebec Canada; 4 Department of Epidemiology, Biostatistics, and Occupational Health, School of Population and Global Health, McGill University, Montreal, QC Canada; 5 Department of Clinical Medicine, Faculty of Health and Medical Sciences, University of Copenhagen, Copenhagen, Denmark; 6 Centre for Mental Health Research, Research School of Population Health, The Australian National University, Canberra, Australia; 7 Department of Mental Health, Johns Hopkins Bloomberg School of Public Health, Baltimore, MD USA

**Keywords:** Suicidal behavior, Self-harm, Suicide attempt, Suicide plans, Suicidal ideation, Mental health, Risk behaviors, Social life, Adolescence

## Abstract

**Objective:**

This study investigated differences in mental health and well-being, risk behaviors, and social life factors among adolescents who experienced different forms of suicidality.

**Methods:**

We examined 18-years-olds in the Danish National Birth Cohort (*N* = 47,852). Suicidality was defined with mutually exclusive categories ranging from no suicidality, self-reported suicide ideation, plans, and attempt as well as hospital-recorded suicide attempt. The proportion of adolescents with self-reported poor mental health and well-being, risk behaviors, and social life factors were compared across forms of suicidality. Sample weights were applied.

**Results:**

Depressive symptoms were reported by 14% (95% CI 13%;14%) of girls with no suicidality, 44% (95% CI 43%;45%) of girls with suicide ideation, and 68% (95% CI 65%;72%) 66% (95% CI 60%;72%) of girls with self-reported suicide attempt or hospital-recorded suicide attempt respectively. Among boys, depressive symptoms were reported by 5% (95% CI 4%;5%) of those with no suicidality, 27% (95% CI 26%;28%) of those with suicide ideation, and 51% (95% CI 45%;57%) and 40% (95% CI 22%;58%) of those with self-reported suicide attempt or hospital-recorded suicide attempt respectively. Likewise, other aspects of poor mental health and well-being gradually increased relative with more severe forms of suicidality, while no notable differences were identified between adolescents with self-reported and hospital-recorded suicide attempt. Similar tendencies were observed for risk behaviors and social life factors.

**Conclusion:**

These findings suggest that adolescents with suicidality, including the large proportion with suicide ideation only, faces challenges across several parameters of mental health and well-being, risk behavior, and social life factors. This emphasizes the need for community-based interventions to identify and support the large group of adolescents experiencing both more and less severe forms of suicidality. Clinicians should prioritize comprehensive psychiatric intervention to address the complex needs of suicidal adolescents effectively.

**Supplementary Information:**

The online version contains supplementary material available at 10.1007/s00787-024-02616-2.

## Introduction

It has been suggested that suicidality constitute a continuum, ranging from suicidal ideation and concrete planning to suicide attempts and suicide [1]. According to international estimates, respectively 20%, 10%, and 7% of 6–21 years old adolescents have self-reported experiences of suicide ideation, suicide plans and suicide attempt [2]. Only a small proportion of these might receive medical attention for their suicidality. Indeed, the majority of suicidal thoughts and behavior occur in the community, i.e., without contact with healthcare professionals in hospitals [3–6]. It has been suggested that mental health factors, such as stress and psychological distress, play a significant role in the development of suicidality [7]. Thus, it seems plausible that severe forms of suicidality might be associated with severe mental health problems. However, sparse evidence exists on potential variations in mental health across the continuum of suicidality. In order to set priorities and design prevention strategies, it is relevant to know how young people experiencing various forms of suicidality during adolescence differs on a broad array of indicators of mental health, risk behavior and social life factors. Further, establishing differences and similarities between adolescents with and without immediate hospital contact following a suicide attempt could improve our understanding of these groups and inform screening and prevention practices [6]. The well-being in late adolescence holds particular significance, since this is a crucial period for shaping one’s future, encompassing important decisions regarding education, career paths, and personal development.

Adolescents without suicidality are generally doing better on mental health outcomes compared to those with suicidal ideation and attempt [8]. Although relatively similar mental health profiles have been noticed for adolescents with suicide ideation and those with suicide plans [9, 10], a higher frequency of eating disorders, body dissatisfaction, depression, anxiety, feeling sad or hopeless, traumatic distress, behavior disorder (OCD, CD, ADHD), alcohol consumption, smoking and substance use has been shown for those adolescent with a suicide attempt [8–10]. However, conflicting findings with no clear patterns have been reported and the current evidence base is limited by being based on small samples and detailed analyses with respect differences by sex and form of suicidality are missing.

Relying on a large Danish community-based sample of 18-years-olds and linked registry data, we aimed to investigate whether factors related to mental health and well-being, risk behaviors, and social life factors differed with respect to forms of suicidality among adolescent girls and boys. In terms of suicidality, we compared those who reported no suicidality, suicide ideation, plans, and attempts as well as those who had been hospitalized with a suicide attempt.

## Methods

### Study design and population

The study population were based on participants in the Danish National Birth Cohort (DNBC). The DNBC is a nested sub-set of the entire population of Danes born during mid-1996 to mid-2003 (*N* = 451,768). Pregnant women were invited to participate in DNBC through their general practitioner (GP) during these years with intentions of terminating pregnancy and insufficient language skills as the only exclusion criteria [11]. Approximately 100,000 pregnancies were enrolled resulting in 96,822 liveborn children [11]. Between 2016 and 2021, when the participants turned 18 years and three months, they received an online questionnaire (DNBC-18). Out of 43,771 eligible girls and 45,434 eligible boys, 27,723 girls (63%) and 20,129 (44%) boys (total: *N* = 47,852, 54%), respectively completed the questionnaire up until the suicidality items (Figure S1, available online). The questionnaire was designed with mandatory responses and if quit early, the completed responses were retained, while unanswered items would be missing. Thus, 26,131 girls (60%) and 18,448 (41%) boys (in total: *N* = 44,579, 50%), completed all indicators of mental health and well-being, risk behaviors, and social life factors used in this study. These two samples (*N* = 47,852 / *N* = 44,579) constituted the central study populations. Data on suicide plans was only available for participants who completed the DNBC-18 after May 2019. Thus, only sub-populations of the former groups (*N* = 19,182 / *N* = 17,829) were available for this form of suicidality. All individuals living in Denmark are issued with a unique personal ID number [12]. This enabled us to link data from the DNBC with register data on hospital-recorded suicide attempt (Table S1).

### Measures

#### Suicidality

Information on self-reported suicidality was obtained from DNBC-18 (Table S2). Participants could answer ‘yes’, ‘no’ and ‘do not know’, when asked whether they ever had experienced suicidal ideation, i.e., “*Have you ever thought about taking your own life (even though you would not do it)?*”, plans, i.e., “*Have you ever had suicide plans (considered methods*,* done preparations)?*”and attempts, i.e., “*Have you ever tried to take your own life?*”. Questions in DNBC-18, which were perceived as particularly sensitive, included ‘do not know’ as a response option. Respectively, 4%, 3%, and 1% of participants replied ‘do not know’ to items regarding suicide ideation, plans and attempts. These were categorized as ‘no’ replies. Participants who responded affirmatively to questions regarding suicide ideation or attempt were also asked about frequency of these events within the last year. These questions were used to assess whether participants recently had experienced suicidality.

Information on hospital-recorded suicide attempts were retrieved from the National Patient Register including both inpatient, outpatient, and emergency room contacts [13]. Suicide attempt was defined as (a) a diagnosis of deliberate self-harm recorded according to the International Classification of Diseases, version 10 (ICD-10) as X60-X84 or where suicide attempt was recorded as the reason for contact. Suicide attempts are under-recorded in Danish hospital settings and we therefore include other contacts, which were likely to be suicide attempts [14]. These were identified as (b) intoxication with specific drugs, (c) a psychiatric diagnosis in combination with intoxication with all drugs and biological substances except alcohol, or d) a psychiatric diagnosis in combination with injuries to the lower forearm (Table S2). This algorithm has previously been applied [15–17]. We only included records of individuals aged 10 years or older as suicide attempts registered in younger individuals could be more likely to reflect accidents or registration error [15]. The admission date in the record was used to determine whether the suicide attempt occurred within a year prior to the completion date of DNBC-18.

A composite measure of form of suicidality was formed with the following mutually exclusive categories: *no suicidality*,* self-reported suicide ideation*,* self-reported suicide attempt*,* and hospital-recorded suicide attempt*. Participant were classified according to their most severe form of suicidality [5]. When analyzing data from the sub-population where information on suicide plans was available, the mutually exclusive categories were: *no suicidality*,* self-reported suicide ideation*,* self-reported suicide plans*,* and suicide attempt (self-reported or hospital recorded)*. Further, an additional measure with the following mutually exclusive categories was constructed to compare adolescents who never, previously, and currently had any form of suicidality: *no suicidality*,* past suicidality (> 1 year prior to completing DNBC-18)*,* and current suicidality (≤ 1 year before completing DNBC-18).*

#### Factors on poor mental health and well-being, risk behavior and social life factors

Measures of poor mental health and well-being consisted of depressive symptoms [18], poor mental well-being [19–21], self-injury within the past year, disordered eating behavior symptoms [22], social anxiety symptoms [23], panic anxiety symptoms [23], externalizing problems [24–26], internalizing problems [24–26], low quality of life [27, 28], and self-assessed health (Table S3). Measures of risk behavior reflected the adolescents willingness to engage in activities or behaviors, which potentially could lead to morbidity, mortality, or other negative consequences [29]. These included alcohol dependence [30, 31], daily smoking, tried cannabis within the last year, tried other drugs within the last year, and sleep deficiency (< 7 h/night) [32]. Social life factors comprised whether adolescents engaged in supportive social relations or had experiences of loneliness, difficulties making friends, and low social abilities [24–26].

### Statistical analyses

All data management and statistical analyses were conducted in SAS version 9.4. Sample weights were calculated based on inverse probability weighting (IPW), using peers from the general population i.e., all Danes born during the same years as DNBC children alive at age 18 years (*N* = 449,288). By allocating larger weight to individuals with a lower probability of participating in DNBC-18, we were able to compensate for differential participation and attrition [33]. In a logistic regression model, we estimated probability of participating in DNBC-18 based on highest parental education, parental income, parental job-status, maternal age at birth, parity, co-living parents, out-of-home placement, any childhood or adolescent psychiatric diagnosis, and any history of parental psychiatric diagnosis (Table 1). All variables were derived from Danish registers and were linked using the unique personal ID number assigned to all residents (Table S1). Weights were calculated separately for boys and girls and truncated to the cut-off value of median + 5*IQR and used in subsequent analyses [34].

In the main analyses, all factors of poor mental health and well-being, risk behavior and social life factors were examined as binary variables. Characteristics based on scales were dichotomized according to previous literature [18, 21, 22, 25–28, 31]. Weighted proportions were calculated for adolescents with no suicidality, self-reported suicide ideation, plans and attempts, and hospital-recorded suicide attempt with their corresponding 95% confidence intervals (95% CI). Similarly, weighted proportions were calculated for no, past, and current suicidality. P-values based on chi-square tests were used to compare: (1) no suicidality vs. suicidal ideation, (2) suicidal ideation vs. self-reported suicide attempt, and (3) self-reported suicide attempt vs. hospital recorded suicide attempt. Similar tests were performed for the sub-group analyses. All analyses were stratified by sex.

### Sensitivity analyses

In sensitivity analyses, measures based on scales were examined as continuous variables and presented as weighted mean scores with corresponding 95% CI for each form of suicidality. P-values based on t-test were used to compare suicidality groups as described in the main analyses. Adolescents replying ‘do not know’ to suicidality might differ from those replying ‘no’. Therefore, we conducted a sensitivity analysis where participants replying ‘do not know’ were categorized as a separate group.

**Table 1:** Characteristics of the general population of children born in mid-1996 to mid-2003 and participants in the 18-year follow-up of the Danish National Birth Cohort (DNBC-18) born during the same period with and without applied sample weights

	Girls			Boys		
	General population (*N* = 218,869)	Unweighted DNBC-18 (*N* = 27,723)	Weighted DNBC-18	General population (*N* = 230,419)	Unweighted DNBC-18 (*N* = 20,129)	Weighted DNBC-18
	*N* (%)	*N* (%)	(%)	*N* (%)	*N* (%)	(%)
**Self-reported background characteristics of DNBC-18 participants**						
**Current educational enrolment** ^**1**^						
High school	N/A^4^	23,175 (84)	(79)	N/A^4^	14 437 (72)	(64)
Vocational education	N/A^4^	1632 (6)	(8)	N/A^4^	3205 (16)	(20)
Other school/education	N/A^4^	1479 (5)	(7)	N/A^4^	1343 (7)	(8)
Not student	N/A^4^	1437 (5)	(7)	N/A^4^	1144 (6)	(8)
**Living with at least one parent** ^**1**^						
Yes	N/A^4^	25,099 (91)	(87)	N/A^4^	18 661 (93)	(89)
No	N/A^4^	2624 (9)	(13)	N/A^4^	1468 (7)	(11)
**Variables used for sample weights**						
**Highest parental education** ^**1**^						
Elementary school	15,466 (7)	657 (2)	(6)	16,582 (7)	426 (2)	(6)
Vocational education	70,462 (32)	7374 (27)	(34)	73,706 (32)	5043 (25)	(34)
High School	12,435 (6)	1318 (5)	(6)	12,960 (6)	944 (5)	(6)
Higher education	100,642 (46)	17,361 (63)	(48)	106,276 (46)	13,032 (65)	(48)
Missing	19,864 (9)	1013 (4)	(7)	20,895 (9)	684 (3)	(7)
**Parental income** ^**1**^						
Q1 (lowest)	53,695 (25)	3508 (13)	(22)	56,577 (25)	2451 (12)	(22)
Q2 (second lowest)	54,038 (25)	6669 (24)	(26)	56,558 (25)	4687 (23)	(26)
Q3 (second highest)	53,770 (25)	8263 (30)	(26)	56,920 (25)	6027 (30)	(26)
Q4 (highest)	54,059 (25)	9266 (33)	(26)	56,748 (25)	6949 (35)	(26)
Missing	3307 (2)	17 (< 0.5)	(< 0.5)	3616 (2)	15 (< 0.5)	(< 0.5)
**Parental job-status** ^**1**^						
In job	159,080 (73)	23,520 (85)	(76)	167,138 (73)	17,149 (85)	(76)
Not in job	53,455 (24)	3922 (14)	(23)	56,359 (24)	2795 (14)	(23)
Absent leave	2968 (1)	260 (1)	(1)	3243 (1)	167 (1)	(1)
Missing	3366 (2)	21 (< 0.5)	(< 0.5)	3679 (2)	18 (< 0.5)	(< 0.5)
**Maternal age at birth** ^**2**^						
≤25	44,719 (20)	3741 (13)	(19)	46,983 (20)	2613 (13)	(19)
26–30	85,625 (40)	11,834 (43)	(39)	89,937 (39)	8573 (43)	(39)
31–36	72,355 (33)	10,204 (37)	(34)	76,654 (33)	7449 (37)	(34)
>36	16,103 (7)	1940 (7)	(8)	16 754 (7)	1484 (7)	(8)
Missing	67 (< 0.5)	4 (< 0.5)	(< 0.5)	91 (< 0.5)	10 (< 0.5)	(< 0.5)
**Parity (number of liveborn children at birth of index child)** ^**2**^						
1	92,249 (42)	12,765 (46)	(43)	97,083 (42)	9504 (47)	(43)
2	79,151 (36)	9688 (35)	(37)	83,500 (36)	6931 (34)	(37)
≥ 3	41,179 (19)	4246 (15)	(18)	43,392 (19)	3019 (15)	(18)
Missing	6290 (3)	1024 (4)	(3)	6444 (3)	675 (3)	(3)
**Co-living parents** ^**1**^						
No	99,166 (45)	10,925 (39)	(47)	101,160 (44)	7158 (36)	(45)
Yes	113,858 (52)	16,784 (61)	(53)	123,068 (53)	12 952 (64)	(55)
Missing	5845 (3)	14 (< 0.5)	(< 0.5)	6191 (3)	19 (< 0.5)	(< 0.5)
**Out-of-home placement** ^**3**^						
Yes	6993 (3)	344 (1)	(3)	7738 (3)	248 (1)	(3)
**Psychiatric diagnosis in adolescent** ^**3**^						
Yes	26,327 (12)	3055 (11)	(13)	28,468 (12)	2047 (10)	(13)
**Parental psychiatric diagnosis** ^**3**^						
Yes	43,425 (20)	3996 (14)	(20)	45,808 (20)	2796 (14)	(20)

^1^Measured the year the adolescent turned 18 years

^2^Measured at birth

^3^Measured up until the adolescent turned 18 years

^4^Not applicable as self-reported information is not available for the general population

## Results

Among 27,723 girls and 20,129 boys in our sample, respectively 84% and 72% were attending high school and 91% and 93% lived with at least one parent (Table 1). A larger proportion of participants had parents with longer educations, higher income level, and were working when compared to the general population of Danes born in the same period as DNBC children who were identified in national register data. After applying sample weights, the distribution of the participants’ socio-demographic characteristics resembled those of peers in the general population. In the sample, 16,740 (58%) girls and 14,225 (69%) boys expressed no suicidality, while 9763 (36%) girls and 5491 (28%) boys had suicide ideation, 854 (4%) girls and 359 (2%) boys had self-reported suicide attempt and 366 (2%) girls and 54 (0.4%) boys had hospital-recorded suicide attempt, respectively.

### Poor mental health and well-being

**Fig. 1:**
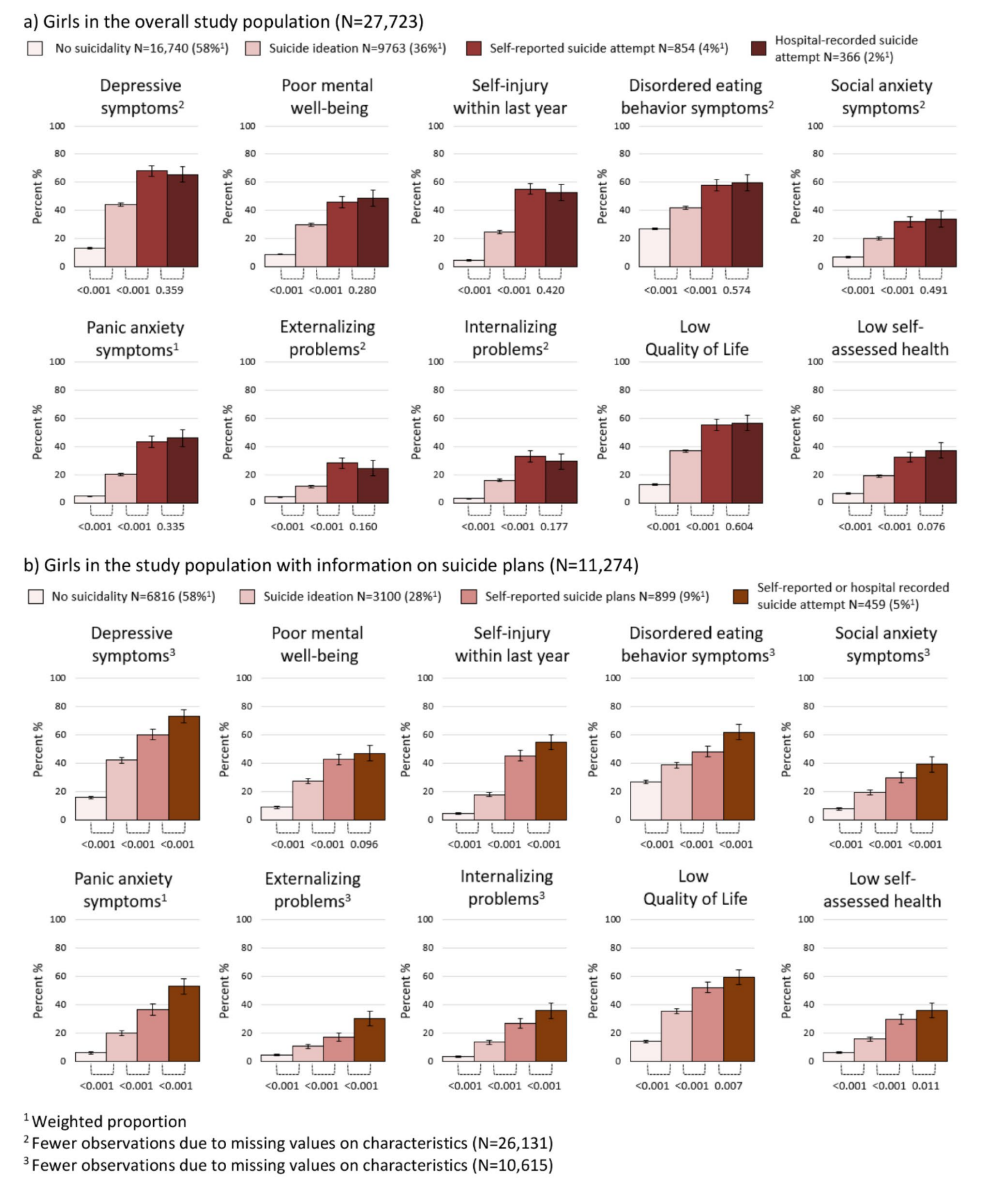
Weighted proportion of poor mental health and well-being in 18-year-old girls according to suicidality, presented with 95% confidence intervals and p-values comparing proportions between groups

Depressive symptoms were reported by 13.5% (95% CI 12.9%;14.1%) of girls with no suicidality, while such symptoms were reported by 44.2% (95% CI 42.1%;45.3%), 68.2% (95% CI 64.5%;71.8%), and 65.6% (95% CI 60.0%;71.3%) of girls with suicide ideation, self-reported suicide attempt, and hospital-recorded suicide attempt, respectively (Fig. 1, Table S4). Similar patterns of higher proportions of other markers for adverse mental health and well-being were seen for the more severe forms of suicidality although no significant differences in proportions were found between self-reported and hospital-recorded suicide attempts for girls. Among girls with information on suicide plans, an increasingly larger proportion likewise reported poor mental health and well-being relative to more severe levels of suicidality (Fig. 1, Table S5). Girls with suicide attempt reported the highest proportions of poor mental health and well-being. However, responses from girls with suicide plans revealed a comparable high prevalence of poor mental well-being. The proportions of self-injury, low quality of life, and low self-assessed health of girls with suicide plans were closer to those of girls with suicide attempt than the levels found for girls with suicide ideation.

**Fig. 2:**
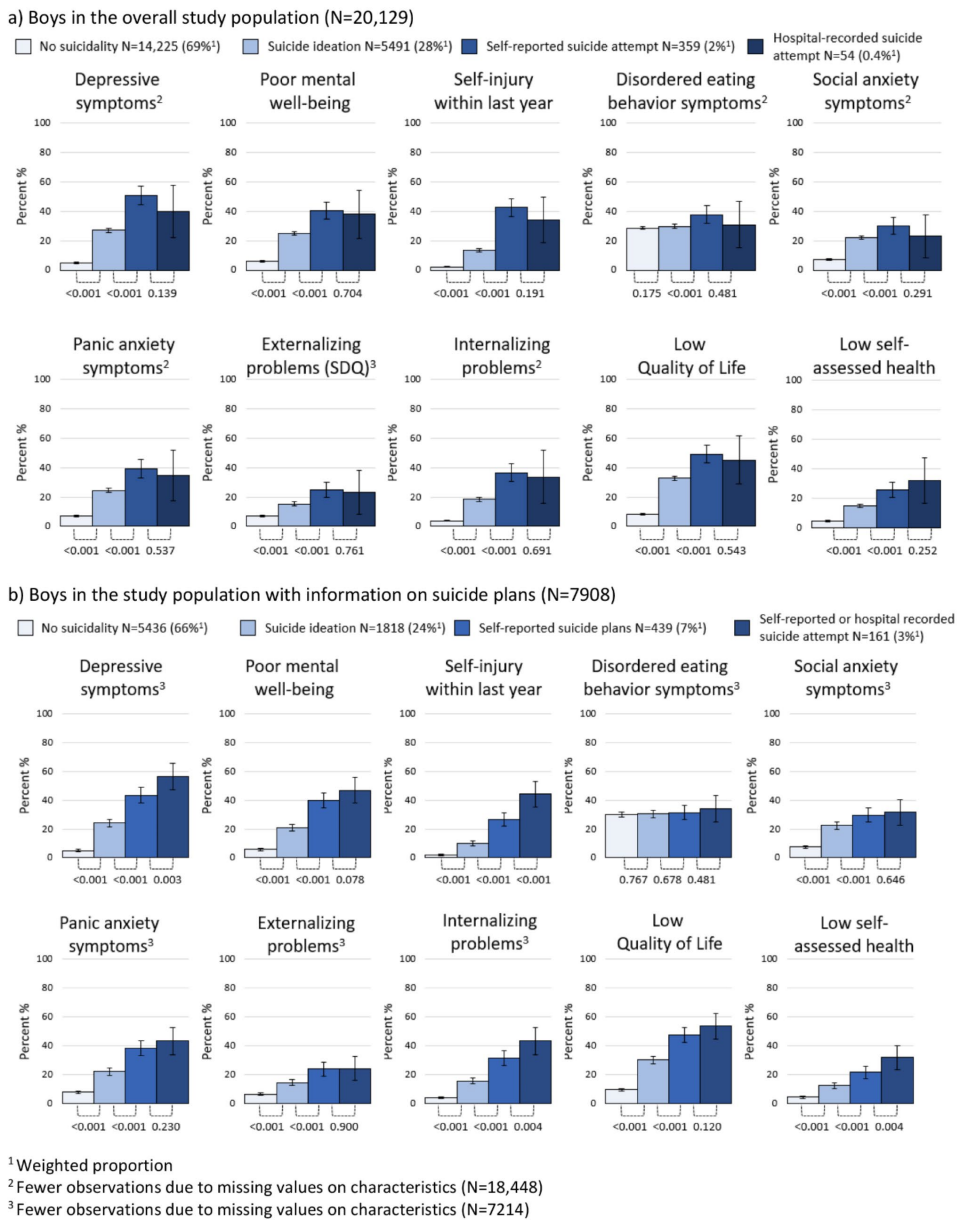
Weighted proportion of poor mental health and well-being in 18-year-old boys according to suicidality, presented with 95% confidence intervals and p-values comparing proportions between groups

Depressive symptoms were reported by 4.7% (95% CI 4.3%;5.2%) of boys with no suicidality, while such symptoms were reported by 26.9% (95% CI 25.5%;28.4%), 50.9% (95% CI 44.6%;57.2%) and 39.9% (95% CI 22.0%;57.8%) of boys with suicide ideation, self-reported suicide attempt, and hospital-recorded suicide attempt, respectively (Fig. 2). Similar patterns were observed across all measures of poor mental health and well-being for boys as were seen for girls, except from disordered eating behavior where boys with no suicidality (28.5%; 95% CI 27.7%;29.4%) and suicide ideation (29.6%; 95% CI 28.1%;31.0%) reported comparable levels. Confidence intervals for self-reported and hospital-recorded suicide attempts were relatively wide, which obscured comparison. Among boys with information on suicide plans, the proportion of those with poor mental health and well-being also increased relative with more severe forms of suicidality. Significant differences were not detected for suicide plans vs. suicide attempts with regard to poor mental well-being, disordered eating behavior, social and panic anxiety, externalizing problems, and low quality of life (Fig. 2).

### Risk behaviors

**Fig. 3:**
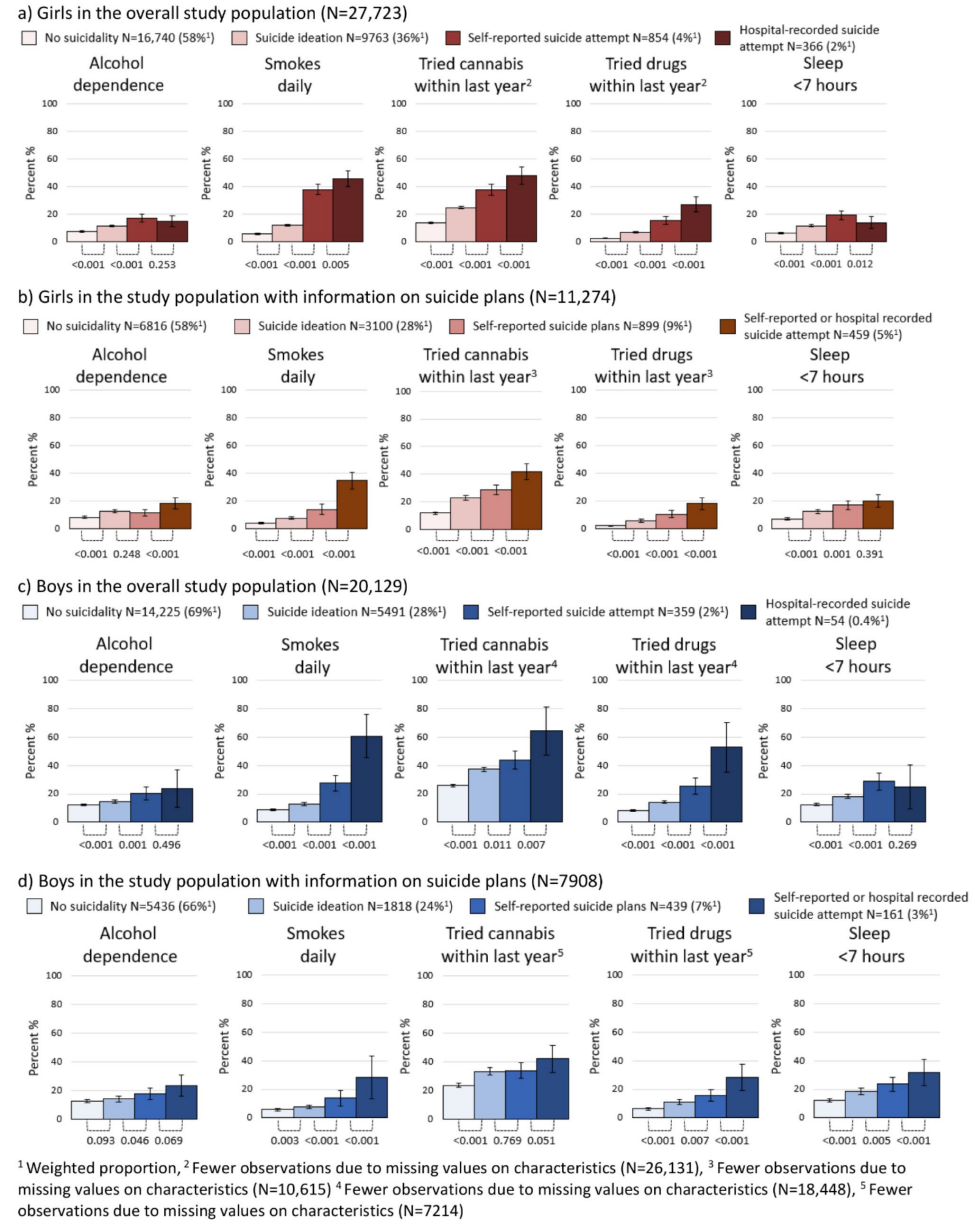
Weighted proportion of risk behavior in 18-year-olds according to suicidality, presented with 95% confidence intervals and p-values comparing proportions between groups

Risk behaviors were more frequent among girls who reported more severe forms of suicidality (Fig. 3). The same was found for the sub-population where information on suicide plans was available. Smoking, cannabis use, and drug use were more common among girls with hospital-recorded suicide attempts when compared to those with self-reported attempts. Similar patterns were observed among boys. A larger share of boys with more severe forms of suicidality reported smoking, drug use, and sleep deficiency, also when including suicide plans plan.

### Social life factors

**Fig. 4:**
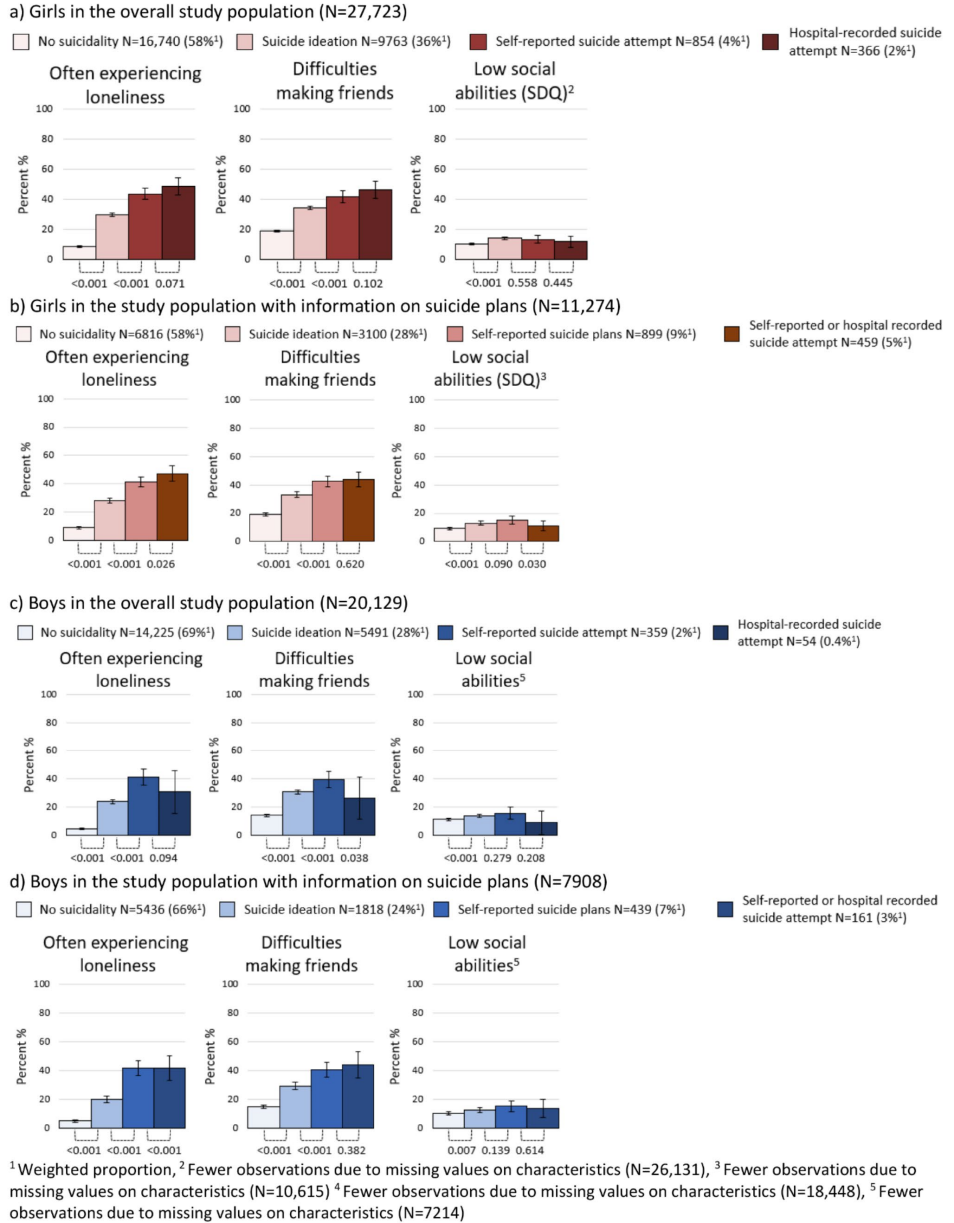
Weighted proportion of social life factors in 18-year-olds according to suicidality, presented with 95% confidence intervals and p-values comparing proportions between groups

Individuals considered to have more severe forms of suicidality more frequently reported loneliness and difficulties making friends (Fig. 4). For both sexes, no notable differences among adolescents with suicidality were observed with regard to low social abilities.

### Past and current suicidality

Respectively, 29% and 22% of 18-year-old girls and boys had experienced some form of suicidality within the last year, while 13% girls and 9% boys had experienced suicidality in the past, but not within the last year (Figure S2). The proportion who reported poor mental health and well-being was highest among adolescents with current suicidality. Nevertheless, adolescents with past suicidality, i.e., more than a year ago, were still doing markedly worse at age 18 years when compared to adolescents who had never experienced any suicidality.

### Sensitivity analyses

Sensitivity analyses of continuous variables revealed similar patterns, although boys with self-reported suicide attempt had significantly higher scores on scales of depressive symptoms, social anxiety, internalizing problems, and quality of life than boys with hospital-recorded suicide attempt (Table S6). For boys, those with suicide attempts were scoring significant worse with regard to depressive symptoms, panic anxiety, externalizing and internalizing problems, and alcohol dependence when compared to those with suicide plans (Table S7). Overall, adolescents who responded ‘do not know’ to suicidality had a higher proportion of poor mental health and well-being, risk behaviors, and negative social factors compared to adolescents who responded ‘no’ to suicidality (Table S8). However, these proportions were still lower than among adolescents reporting suicidal ideation (Table S4, Table S8).

## Discussion

When comparing self-reported and hospital-recorded data on suicidality, we found that the proportion of adolescents experiencing poor mental health and well-being seemed to increase relative with more severe forms of suicidality i.e., from no suicidality to suicide ideation, plans and attempt in both girls and boys. About a quarter of adolescents had experienced suicide ideation and the prevalence of poor mental health and well-being in this group was 2-4-fold higher compared to those with no suicidality. Further, adolescents with suicide plans resembled those with suicide attempt with respect to some characteristics, such as poor mental well-being. In general, the highest proportions of poor mental health and well-being, risk behavior and social life factors were most common among 18-years-olds who experienced a suicide attempt in adolescence, regardless of whether the suicide attempt was self-reported or hospital-recorded. Yet, smoking, use of cannabis and other drugs were found to be more frequent among adolescents with hospital-recorded suicide attempt versus those who self-reported suicide attempt.

These findings add important information to the limited literature on the characteristics of youth with different forms of suicidality. Indeed, although differences in mental health and well-being, risk behavior and social life factors with respect to different forms of suicidality have been suggested, no major differences or a clear pattern has previously been observed [8–10]. Two of the referenced studies adjusted for sex, age, and ethnicity, and sex and socioeconomic position respectively in their analyses [8, 10]. Sex and age were indirectly adjusted for in our study by comparing 18-year-old girls and boys separately. We opted to conduct unadjusted analyses as the aim was to investigate how adolescents who experienced different forms of suicidality were doing at age 18 years rather than establish causal associations. Discrepancies in findings could also be attributed to the larger sample size with older adolescents and the different measures of characteristics in our study. Additionally, previous studies mostly investigated characteristics/factors occurring during childhood and early adolescence rather than adolescents’ current or recent state or experiences, which makes it difficult to directly compare our finding and those from previous studies.

The emotional turmoil and psychological distress associated with experiences of suicide ideation, plans and attempt may impede adolescents abilities to envision a positive future and focus on academic pursuits, engage in healthy relationships, and other activities, which contribute to personal growth. Support for mental health problems, including suicidality, during this formative age is crucial for mitigating long-term consequences. Poor mental health and well-being were particularly common among those experiencing current suicidality at age 18 years. However, it is also concerning that individuals who no longer experienced suicidality by the age of 18 years still faced mental health challenges, considering the high risk of repetition of self-harm and suicidality [35–37].

Different theoretical frameworks for suicidal behavior exist and some suggest that a complex interplay of factors related to psychiatric illness, stress, psychological pain/distress, cognitive vulnerability, and personality traits may lead to elevated risk of suicidality [7]. Hopelessness and psychological pain are suggested to be crucial components that motivates suicidality [38]. Our findings support this perspective, as we found that poor mental health and well-being, which could cause such pain and hopelessness, were more common among those with suicidality compared to those without. Ideation-to-action theories of suicide have emphasized the importance of ‘suicide capability’ in the progression from suicidal thought to suicide attempts [8, 38]. Factors such as drug use and self-injury have been used as proxies for acquired ‘suicide capability’ and we accordingly found that these factors were substantially more prevalent among adolescents with suicide attempt compared to those with ideation only.

In treatment of adolescents with suicidality, clinicians should be mindful of its co-existence with several other adverse aspects of mental health in their assessments and explore comprehensive treatment strategies rather than solely concentrating on a single concern. While it is relatively rare that suicide ideation escalates into more severe forms of suicidality, this sizable group appears to struggle significantly across various factors, while stigma and lack of literacy regarding suicide prevention may hinder adolescents from seeking support [39]. Given that only a minority of adolescents seek help for suicidal thoughts and behavior [40], universal school-based interventions might improve detection of at-risk individuals [41, 42].

Suicide attempts with hospital-contact constitute the tip of the iceberg, as the majority of suicide attempts does not lead to hospital contact [4, 5]. Suicide attempts that are not leading to hospital contact, might be less serious attempts where the methods used are less lethal so that physical injuries do not require medical attention [4]. We found that the group with community-based suicide attempts had comparable levels of poor mental health and well-being to those with hospital-recorded suicide attempts. This emphasizes the importance of identifying and providing support to the larger group of individuals with a community-based suicide attempt. Although some adolescents might have presented to primary care providers, it is concerning that as many as 68% and 51% of girls and boys with a self-reported suicide attempt also reported depressive symptoms. Boys with self-reported suicide attempts even had a significantly higher score on the depressive symptoms scale when compared to boys with hospital-recorded suicide attempts. Future research should focus on establishing whether adolescents with community-based suicide attempt receive help for their psychological problems.

### Strengths and limitations

Strengths of this study include a large study population, sample weights based on national data to reduce bias of differential participation and attrition, multiple measures of self-reported suicidality linked to hospital-recorded suicide attempt, in addition to numerous self-reported measures of mental health and well-being, risk behavior, and social life factors. The DNBC-18 was pilot tested by young people in focus groups to ensure that the questions was understood as intended.

Limitations in this study should also be acknowledged; (1) Although the response rate of the DNBC-18 was comparable to other cohort studies [43], the initial selection into the cohort and the 54% response rate may have led to selection bias. We tried to minimize this by using sample weights. It is possible that adolescents who participated and reported experiencing suicidality might have been doing better than non-participants with suicidality, thus, rending our estimates conservative, although we applied sample weights based on psychiatric diagnosis to adjust for this; (2) suicide was not included as a measure, as death precluded from participating in DNBC-18. However, only few suicides (*n* = 19, 0.02%) were observed before age 18 years among participants in the DNBC; (3) it would have been desirable to have had information regarding the exact timing of self-reported suicidality and characteristics as this would have improved the precision of our estimates. In additional analyses, we distinguished between suicidality occurring within the past year and events, which occurred more than a year ago. These analyses revealed that the majority, i.e. 29% girls and 22% boys had experienced suicidality within the past year while 13% girls and 9% boys no longer experienced suicidality; (4) the number of hospital-recorded suicide attempts was relatively low, particularly among boys. This implied greater uncertainty, i.e. wider confidence intervals, which prevented identification of differences between forms of suicidality; (5) misclassifications may have affected the results. The identification of suicide attempt in hospital-records did not necessarily include a direct assessment of suicidal intentions. Including likely suicide attempt, such as those with a poisoning, may have resulted in misclassification of accidents as suicide attempts. However, among those identified as having a hospital-recorded suicide attempt, 78% of girls and 54% of boys also self-reported a suicide attempt, and 95% of girls and 72% of boys self-reported suicide ideation [5]. Conversely, some adolescents with self-reported suicide attempt, may have presented to the hospital without being recorded as such. The question regarding suicide ideation contained vague phrases, i.e. “*even though you would not do it”*. Nevertheless, notable differences were observed between adolescents with suicide ideation only and those without suicidality [8]. We adopted a conservative approach by grouping the small group of adolescents replying ‘do not know’ to suicidality into the large ‘no suicidality’ group. However, these groups differed on several characteristics and ‘do not know’ answers may have reflected suicidality (Table S8). We categorized characteristics to reflect the proportion of adolescents who were unwell from a clinical perspective and to enhance interpretability. This simplification comes at the cost of level of detail and may lead to imprecise characterizations. For instance, for some characteristics we opted for a less severe category, such as ‘tried cannabis within the last year’ instead of ‘cannabis dependency’ to ensure a reasonable number of observations in each group. When characteristics were analyzed on continuous scales, more comparisons of characteristics in adolescents with different forms of suicidality were significantly different. However, these distinctions may carry less clinical significance in the broader context.

## Conclusion

In conclusion, findings from this study indicate that adolescents who experienced suicidality, even the large proportion reporting suicidal ideation only, faces challenges of poor mental health and well-being, risk behaviors and negative social life factors. Compared to those with hospital-recorded suicide attempt, adolescents with community-based suicide attempt have similar symptoms of poor mental health and well-being. This emphasizes the need to identify and provide psychiatric help to this ‘hidden’ group not immediately presenting at the hospital. Clinicians must treat suicidal thoughts and behaviors in adolescents with seriousness and address the additional challenges these individuals may be struggling with.

## Electronic supplementary material

Below is the link to the electronic supplementary material.

Supplementary Material 1

## Data Availability

According to European law (General Data Protection Regulation), data containing potentially identifying or sensitive personal information are restricted. However, for academic research, data can be available subsequent to approval. Any request for DNBC data needs to follow the outlined procedures, see: https://www.dnbc.dk/access-to-dnbc-data. For access to the underlying person-level data in Danish registers, researchers need to apply to the Danish Health Data Authority and Statistics Denmark. The programming codes used on DNBC and register data in this study are available by contacting the corresponding author.
