# Supplementary material for: Mental health, risk behaviors, and social life factors in relation to adolescents’ suicide ideation, plans and attempt
Source: Eur Child Adolesc Psychiatry. 2024 Nov 15;34(6):1945–58. doi: 10.1007/s00787-024-02616-2 (PMC12198280; doi:10.1007/s00787-024-02616-2)
Supplement: Supplementary file 1 — Supplementary Material 1 [file 787_2024_2616_MOESM1_ESM.docx]

**Supplemental Material**

**Table S1**. Data sources for register-based variables included in the analyses.

| **Variable** | **Register** |
| --- | --- |
| Highest parental education | The Education Register^1^ |
| Parental income | The Income Statistics Register^2^ |
| Parental job status | The Employment Classification Module^3^ |
| Maternal age at birth | The Medical Birth Register^4^ |
| Parity |  |
| Sex | The Civil Registration System^5^ |
| Co-living parents |  |
| Out-of-home placement | The Register of Support for Children and adolescents^6^ |
| Psychiatric diagnosis in adolescent | The National Patient Register^7^ and the Psychiatric Central Research Register^8^ |
| Parental psychiatric diagnosis |  |
| Hospital-recorded suicide attempt (see Supplementary Table 2) |  |
| ^1^Jensen, V. M. & Rasmussen, A. W. Danish education registers. Scand. J. Public Health 39, 91–94 (2011). ^2^Baadsgaard, M. & Quitzau, J. Danish registers on personal income and transfer payments. Scand. J. Public Health 39, 103–105 (2011). ^3^Petersson, F., Baadsgaard, M. & Thygesen, L. C. Danish registers on personal labour market affiliation. Scand. J. Public Health 39, 95–98 (2011). ^4^Bliddal, M., Broe, A., Pottegård, A., Olsen, J. & Langhoff-Roos, J. The Danish Medical Birth Register. Eur. J. Epidemiol. 33, 27–36 (2018). ^5^Pedersen, C. B. The Danish Civil Registration System. Scand. J. Public Health 39, 22–25 (2011). ^6^https://www.dst.dk/da/Statistik/dokumentation/statistikdokumentation/anbringelser-af-boern-og-unge/indhold ^7^Lynge, E., Sandegaard, J. L. & Rebolj, M. The Danish National Patient Register. Scand. J. Public Health 39, 30–33 (2011). ^8^Mors, O., Perto, G. P. & Mortensen, P. B. The Danish Psychiatric Central Research Register. Scand. J. Public Health 39, 54–57 (2011). | |

**Table S2.** Definitions of self-reported and register-based suicidality.

| **Measure Questions regarding suicidality in the DNBC 18** | | **Response options** |
| --- | --- | --- |
| Suicide ideation | Have you ever thought about taking your own life (even though you would not do it)? | Yes, No and Do not know |
| Suicide plans^1^ | Have you ever had suicide plans (considered methods, done preparations)? | Yes, No and Do not know |
| Suicide attempt | Have you ever tried to take your own life? | Yes, No and Do not know |
| **Operational definition of hospital-recorded suicide attempt** | | |
| Hospital-recorded suicide attempt | A hospital contact where either:  a) a main or supplementary diagnosis of X60-X84 was recorded according to the International Classification of Diseases, version 10 (ICD-10) or where reason of contact code was listed as “suicide attempt”.  b) a main diagnosis of accidental intoxication with weak analgesic drugs, antiepileptics, sleeping pills, antiparkinsonian drugs, psychotropics, and carbon monoxide (ICD-10: T39, T40 except T40.1, T42, T43, T58) was recorded.  c) a main diagnosis of a psychiatric disorder (ICD-10: F00-F99) was recorded in combination with a supplementary diagnosis of intoxication with drugs and biological substances (ICD-10: T36-T50, T52-T60).  d) a main diagnosis of a psychiatric disorder (ICD-10: F00-F99) was recorded in combination with a supplementary diagnosis of injuries to the lower forearm (ICD-10: S51, S55, S59, S61, S65, S69). | |
| ^1^ Only available for participants replying to DNBC-18 after May 2019 (N=19,182). | | |

**Table S3.** Description of instruments used for the assessment of mental health and well-being, risk behaviors and social life factors in 18-year-olds.

| **Characteristic** | **Scale** | **Description** | **Scale score and cut-offs** |
| --- | --- | --- | --- |
| **Mental health and well-being** | | | |
| **Depressive symptoms^1^** | The Major Depression Inventory (MDI) | Compiled from 12 items referring to the past two weeks.  Response options ranging from 0=at no time to 5=all the time | Overall score ranged from 0-50.  Scores ≥20 were considered as depressive symptoms. |
| **Poor mental well-being^2,3^** | Short Warwick-Edinburgh Mental Well-Being Scale (SWEMWBS) | Compiled from 7 items referring to the past two weeks.  Response options ranging from 1=always to 5=never | Overall score ranged from 7-35.  Scores ≤20 were considered as poor mental well-being. |
| **Self-injury within the last year** |  | Based on the question ‘How often have you intentionally harmed or hurt yourself within the last year’. Response options were ‘at no time, ‘one time’, ‘2-5 times’, ‘6-10 times’, ‘more than 10 times’ and ‘do not know’. | A reply of ‘one time – more than 10 times’ was considered as self-injury within the last year.  A ‘do not know’-reply (3%) was considered as ‘no self-injury’ together with ‘at no time’. |
| **Disordered eating behavior symptoms^4^** |  | Compiled from 22 items regarding height and weights and current symptoms of anorexia, bulimia, binge eating, and other disordered eating behaviors. | At least monthly symptoms of anorexia, bulimia, binge eating, or other disordered eating behaviors were considered as disordered eating behavior symptoms. |
| **Social anxiety^5^** | Sub-scale of The Spence Children’s Anxiety Scale (SCAS) | Compiled from 6 items referring to how often one experiences symptoms.  Response options ranged from 0=never to 3=always. | Overall score ranged from 0-18. Cut-off points were identified as the value, which 10% of the participants had scored above. Scores of ≥13 (girls) and ≥9 (boys) were considered as cut-off points for symptoms of social anxiety. |
| **Panic anxiety^5^** | Sub-scale of The Spence Children’s Anxiety Scale (SCAS) | Compiled from 9 items referring to how often one experiences symptoms.  Response options ranged from 0=never to 3=always. | Overall score ranged from 0-27. Cut-off points were identified as the value, which 10% of participants had scored above. Scores of ≥10 (girls) and ≥4 (boys) were considered as cut-off points for symptoms of panic anxiety. |
| **Externalizing problems^6-8^** | Sub-scale of Strengths and Difficulties Questionnaire (SDQ) | Compiled from 10 items from the subscales for conduct problems and hyperactivity/inattention reflecting externalizing problems. Response options ranging from 0=not true to 3=certainly true. Reducing subscales to dimensions of externalizing problems have been shown to be advantageous in low-risk samples. | Overall score ranged from 0-20. Cut-off points were identified as the value, which 10% of participants had scored above. Scores of ≥10 (girls) and ≥9 (boys) were considered as cut-off points for externalizing problems. |
| **Internalizing problems^6-8^** | Sub-scale of SDQ | Compiled from 10 items from the subscales for emotional symptoms and peer relationship problems reflecting internalizing problems. Response options ranging from 0=not true to 3=certainly true. Reducing subscales to dimensions of internalizing problems have been shown to be advantageous in low-risk samples. | Overall score ranged from 0-20. Cut-off points were identified as the value, which 10% of participants had scored above. Scores of ≥11 (girls) and ≥8 (boys) were considered as cut-off points for internalizing problems. |
| **Low quality of life^9,10^** | Cantril’s Ladder | Based on the question ‘How satisfied are you currently with your life? Indicate satisfaction on a scale from 0-10, where 10 is the best possible life and 0 is the worst imaginable life’ | Overall scores ranged from 0-10. Participants who scored ≤5 were considered as having low quality of life. |
| **Self-assessed health** |  | Based on the question ‘How would you describe your overall health’.  Response options were ‘outstanding’, ‘very good’, ‘less good, and ‘bad’. | Participants who answered ‘bad’ were considered as having poor self-assessed health. |
| **Risk behavior** | | | |
| **Alcohol dependence^11,12^** | Alcohol Use Disorders Identification Test (AUDIT). | Compiled from 8 items referring to the last year. Response options regarding experiences with alcohol ranging from 0=never to 4=daily or almost daily. Response options regarding quantity of alcohol consumed when drinking ranging from 0=1-2 to 4=more than 10. | Overall scores ranged from 0-40.  Participants who scored ≥15 were considered to have an alcohol dependence. Adolescents who previously had reported abstinence were not administered the AUDIT and were considered to have ‘no alcohol dependence’. |
| **Smokes daily** |  | Based on the question ‘How often do you currently smoke cigarettes?’ with response options ‘every day’, ‘at least once a month (not every week)’, ‘at least once a week (not every day)’, and ‘I do not smoke’. | An ‘every day’-reply was considered as daily smoking. |
| **Tried cannabis within the last year / Tried other drugs within the last year** |  | Based on the question ‘Have you tried any of the following drugs within the past year’. Response options were cannabis, amphetamine, ecstasy/MDA/, fantasy, coke, LSD, opioids, mushrooms, and inhaling of nitrous oxide or solvents | Substance use within the last year were divided into two variables; one for ‘cannabis’ (yes/no) and one for ‘other drugs’ (yes/no). |
| **Sleep deficiency (<7 hours/night)^13^** |  | Based on the 2 question ‘At what time do you typically go to sleep on weekdays’ and ‘When do you typically wake up or are woken up on weekdays’. Sleep duration was calculated as the time difference between reporting going to sleep and waking up on average weekdays. | Participants with <7 hours sleep/night were considered as having sleep deficiency, based on Centre for Disease Control, USA, recommends. A ‘do not know’-reply (3%) was considered as ‘no sleep deficiency’ together with at least 7 hours sleep/night. |
| **Social life factors** | | | |
| **Loneliness** |  | Based on the question ‘How often do you feel lonely’.  Response options were ‘never’, ‘once in a while’, ‘often’, ‘very often’ and ‘do not know’. | Participants who responded ‘often/very often’-were considered as often experiencing loneliness.  Those who answered ‘do not know’ (3%) were considered to have ‘no loneliness’ as were those who answered ‘never/once in a while’. |
| **Difficulties making friends** |  | Based on the question ‘Is it easy or difficult for you to make new friends’.  Response options were ‘very easy’, ‘easy’, ‘difficult, ‘very difficult’ and ‘do not know’. | Participants who responded ‘difficult/very difficult’ were considered as having difficulties making friends. Those who answered ‘do not know’ (9%) were considered to have ‘no difficulties making friends’ as were those who answered ‘easy/very easy’. |
| **Low social abilities^6-8^** | Sub-scale of SDQ | Compiled from 5 items from the subscale pro-social behavior. Response options ranging from 0=not true to 3=certainly true. | Overall score ranged from 0-10. Cut-off points were identified as the value, which 10% of participants had scored above. Scores of ≤6 (girls) and ≤5 (boys) were considered as cut-off points for low social abilities. |
| ^1^Bech, P., Timmerby, N., Martiny, K., Lunde, M. & Soendergaard, S. Psychometric evaluation of the Major Depression Inventory (MDI) as depression severity scale using the LEAD (Longitudinal Expert Assessment of All Data) as index of validity. BMC Psychiatry 15, 190 (2015). ^2^Koushede, V. et al. Measuring mental well-being in Denmark: Validation of the original and short version of the Warwick-Edinburgh mental well-being scale (WEMWBS and SWEMWBS) and cross-cultural comparison across four European settings. Psychiatry Res. 271, 502–509 (2019). ^3^Ng Fat, L., Scholes, S., Boniface, S., Mindell, J. & Stewart-Brown, S. Evaluating and establishing national norms for mental wellbeing using the short Warwick–Edinburgh Mental Well-being Scale (SWEMWBS): findings from the Health Survey for England. Qual. Life Res. 26, 1129–1144 (2017). ^4^Micali, N. et al. Adolescent Eating Disorders Predict Psychiatric, High-Risk Behaviors and Weight Outcomes in Young Adulthood. J. Am. Acad. Child Adolesc. Psychiatry 54, 652-659.e1 (2015).  ^5^Spence, S. H., Barrett, P. M. & Turner, C. M. Psychometric properties of the Spence Children’s Anxiety Scale with young adolescents. J. Anxiety Disord. 17, 605–625 (2003). ^6^Goodman, R., Meltzer, H. & Bailey, V. The strengths and difficulties questionnaire: A pilot study on the validity of the self-report version. Eur. Child Adolesc. Psychiatry 7, 125–130 (1998). ^7^Goodman, A., Lamping, D. L. & Ploubidis, G. B. When to Use Broader Internalising and Externalising Subscales Instead of the Hypothesised Five Subscales on the Strengths and Difficulties Questionnaire (SDQ): Data from British Parents, Teachers and Children. J. Abnorm. Child Psychol. 38, 1179–1191 (2010). ^8^Scheel Rasmussen, I., Strandberg-Larsen, K., Overbeck, G. & Wilson, P. A critical examination of Danish norms for the Strengths and Difficulties Questionnaire (SDQ). Nord. J. Psychiatry 1–6 (2023) doi:10.1080/08039488.2023.2250316. ^9^Levin, K. A. & Currie, C. Reliability and Validity of an Adapted Version of the Cantril Ladder for Use with Adolescent Samples. Soc. Indic. Res. 119, 1047–1063 (2014). ^10^Holstein, B. E., Trab Damsgaard, M., Rich Madsen, K. & Rasmussen, M. Persistent social inequality in low life satisfaction among adolescents in Denmark 2002–2018. Child. Youth Serv. Rev. 116, 105097 (2020). ^11^Saunders, J. B., Aasland, O. G., Babor, T. F., De La Fuente, J. R. & Grant, M. Development of the Alcohol Use Disorders Identification Test (AUDIT): WHO Collaborative Project on Early Detection of Persons with Harmful Alcohol Consumption-II. Addiction 88, 791–804 (1993). ^12^Scoring the Audit. https://auditscreen.org/about/scoring-audit/. ^13^Paruthi, S. et al. Recommended Amount of Sleep for Pediatric Populations: A Consensus Statement of the American Academy of Sleep Medicine. J. Clin. Sleep Med. 12, 785–786 (2016). | | | |

**Table S4.** Weighted proportion of characteristics in 18-year-olds according to suicidality, (mutually exclusive categories defined as no suicidality, self-reported suicide ideation, self-reported suicide attempt, and hospital-recorded suicide attempt) presented with 95% confidence intervals (the numbers behind illustrations in Figure 1-4).

|  | **Girls (27,723 / 26,131)^1,2^** | | | | **Boys (N=20,129 / 18,448)^1,2^** | | | |
| --- | --- | --- | --- | --- | --- | --- | --- | --- |
|  | **Self-reported** | | | **Hospital recorded** | **Self-reported** | | | **Hospital recorded** |
|  | **No suicidality**  **N=16,740**  **% (CI 95%)** | **Suicide ideation**  **N=9763**  **% (CI 95%)** | **Suicide attempt**  **N=854**  **% (CI 95%)** | **Suicide**  **Attempt**  **N=355**  **% (CI 95%)** | **No suicidality**  **N=14,225**  **% (CI 95%)** | **Suicide ideation**  **N=5491**  **% (CI 95%)** | **Suicide attempt**  **N=359**  **% (CI 95%)** | **Suicide**  **Attempt**  **N=54**  **% (CI 95%)** |
| **Mental health and well-being** | | | | | | | | |
| **Depressive symptoms^2^** | 13.5 (12.9;14.1) | 44.2 (43.1;45.3) | 68.2 (64.5;71.8) | 65.6 (60.0;71.3) | 4.7 (4.3;5.2) | 26.9 (25.5;28.4) | 50.9 (44.6;57.2) | 39.9 (22.0;57.8) |
| **Poor mental well-being^1^** | 8.9 (8.4;9.4) | 30.0 (29.0;31.0) | 45.8 (42.0;49.6) | 48.8 (43.1;54.6) | 6.0 (5.5;6.5) | 24.9 (23.5;26.3) | 40.5 (34.7;46.3) | 38.1 (21.8;54.4) |
| **Self-injury within the last year^1^** | 4.7 (4.3;5.0) | 24.6 (23.7;25.6) | 55.3 (51.5;59.0) | 53.0 (47.2;58.8) | 2.2 (1.9;2.5) | 13.4 (12.3;14.5) | 42.6 (36.7;48.5) | 34.2 (18.6;49.8) |
| **Disordered eating behavior symptoms^2^** | 26.9 (26.2;27.7) | 41.9 (40.8;43.0) | 58.0 (54.1;62.0) | 59.7 (53.7;65.6) | 28.5 (27.7;29.4) | 29.6 (28.1;31.0) | 37.7 (31.6;43.8) | 30.8 (15.0;46.7) |
| **Social anxiety^2^** | 6.9 (6.4;7.4) | 20.1 (19.1;21.0) | 32.0 (28.2;35.8) | 33.9 (28.0;39.7) | 7.3 (6.8;7.9) | 22.2 (20.8;23.5) | 30.2 (24.3;36.0) | 23.0 (8.3;37.8) |
| **Panic anxiety^2^** | 4.7 (4.3;5.1) | 20.2 (19.2;21.1) | 43.2 (39.2;47.2) | 46.0 (39.9;52.1) | 7.1 (6.5;7.6) | 24.4 (23.0;25.8) | 39.1 (33.0;45.3) | 34.7 (17.3;52.1) |
| **Externalizing problems^2^** | 4.0 (3.7;4.4) | 11.5 (10.7;12.3) | 28.1 (24.4;31.9) | 24.4 (19.0;29.9) | 7.0 (6.4;7.5) | 15.4 (14.2;16.6) | 24.9 (19.5;30.3) | 23.0 (8.0;37.9) |
| **Internalizing problems^2^** | 3.1 (2.8;3.4) | 15.8 (14.9;16.7) | 33.0 (29.1;36.9) | 29.3 (23.7;34.9) | 3.9 (3.4;4.3) | 18.3 (17.1;19.6) | 36.5 (30.4;42.7) | 33.7 (15.7;51.8) |
| **Low quality of life^1^** | 13.0 (12.5;13.6) | 36.7 (35.6;37.8) | 55.3 (51.5;59.1) | 56.7 (51.0;62.5) | 8.5 (8.0;9.1) | 32.8 (31.3;34.2) | 49.1 (43.2;55.0) | 45.2 (29.0;61.3) |
| **Self-assessed health^1^** | 6.6 (6.2;7.0) | 19.1 (18.2;19.9) | 32.5 (28.9;36.1) | 37.2 (31.6;42.8) | 4.5 (4.1;4.9) | 14.6 (13.5;15.7) | 25.5 (20.4;30.6) | 32.1 (16.8;47.3) |
| **Risk behavior** | | | | | | | | |
| **Alcohol dependence^1^** | 7.4 (7.0;7.9) | 11.4 (10.6;12.1) | 17.3 (14.3;20.2) | 14.9 (11.0;18.8) | 12.5 (11.9;13.1) | 14.6 (13.5;15.6) | 20.2 (15.5;25.0) | 23.8 (10.7;36.9) |
| **Smokes daily^1^** | 5.8 (5.3;6.2) | 11.9 (11.2;12.7) | 37.9 (34.1;41.7) | 45.6 (39.8;51.4) | 8.7 (8.1;9.3) | 12.8 (11.7;13.9) | 27.7 (22.2;33.1) | 60.6 (45.6;75.7) |
| **Tried cannabis within the last year^2^** | 13.5 (12.9;14.1) | 24.8 (23.8;25.8) | 37.7 (33.8;41.7) | 48.1 (42.0;54.2) | 25.9 (25.1;26.8) | 37.3 (35.8;38.9) | 43.8 (37.6;50.1) | 64.2 (47.5;80.9) |
| **Tried drugs within the last year^2^** | 2.4 (2.2;2.7) | 7.0 (6.4;7.7) | 15.5 (12.5;18.5) | 27.1 (21.6;32.6) | 8.2 (7.6;8.7) | 14.3 (13.2;15.4) | 25.7 (20.0;31.3) | 52.9 (35.3;70.4) |
| **Sleep deficiency (<7 hours/night)^2^** | 6.2 (5.8;6.6) | 11.5 (10.7;12.3) | 19.2 (15.9;22.4) | 13.9 (9.8;18.0) | 12.5 (11.8;13.2) | 18.3 (17.0;19.5) | 28.6 (22.9;34.4) | 24.8 (9.2;40.4) |
| **Social life factors** | | | | | | | | |
| **Loneliness^1^** | 8.4 (7.9;8.8) | 29.5 (28.5;30.5) | 43.6 (39.8;47.4) | 48.6 (42.8;54.4) | 4.6 (4.2;5.1) | 24.0 (22.7;25.3) | 41.5 (35.7;47.4) | 30.8 (15.8;45.9) |
| **Difficulties making friends^1^** | 19.0 (18.3;19.7) | 34.5 (33.4;35.5) | 41.8 (38.0;45.5) | 46.3 (40.5;52.1) | 14.2 (13.6;14.9) | 30.9 (29.5;25.8) | 39.8 (33.9;45.6) | 26.6 (11.7;41.5) |
| **Low social abilities^2^** | 10.4 (9.9;11.0) | 14.0 (13.2;14.8) | 13.3 (10.7;15.9) | 11.8 (8.1;15.5) | 11.3 (10.7;11.9) | 13.9 (12.8;15.0) | 15.8 (11.3;20.4) | 9.1 (0.8;17.5) |
| ^1^ N=27,723 (girls), N=20,129 (boys)  ^2^ N=26,131 (girls), N=18,448 (boys) | | | | | | | | |

**Table S5.** Weighted proportion of characteristics in 18-year-olds according to suicidality, (mutually exclusive categories defined as no suicidality, self-reported suicide ideation, self-reported suicide plans, and self-reported or hospital-recorded suicide attempt) presented with 95% confidence intervals (the numbers behind illustrations in Figure 1-4).

|  | **Girls (11,274 / 10,615)^1,2^** | | | | **Boys (N=7908 / 7214)^1,2^** | | | |
| --- | --- | --- | --- | --- | --- | --- | --- | --- |
|  | **Self-reported** | | | **Hospital recorded/**  **self-reported** | **Self-reported** | | | **Hospital recorded/**  **self-reported** |
|  | **No suicidality N=6816**  **% (CI 95%)** | **Suicide ideation**  **N=3100**  **% (CI 95%)** | **Suicide plans N=899**  **% (CI 95%)** | **Suicide**  **Attempt N=459**  **% (CI 95%)** | **No suicidality N=5436**  **% (CI 95%)** | **Suicide ideation**  **N=1818**  **% (CI 95%)** | **Suicide plans N=439**  **% (CI 95%)** | **Suicide**  **Attempt N=161**  **% (CI 95%)** |
| **Mental health and well-being** | | | | | | | | |
| **Depressive symptoms^2^** | 15.7 (14.7;16.8) | 42.2 (40.2;44.2) | 60.2 (56.5;63.9) | 73.3 (68.6;78.1) | 5.0 (4.3;5.8) | 24.1 (21.6;26.6) | 43.4 (38.0;48.8) | 56.4 (47.1;65.8) |
| **Poor mental well-being^1^** | 8.9 (8.2;9.7) | 27.4 (25.6;29.2) | 42.7 (39.0;46.4) | 47.2 (42.0;52.4) | 5.8  (5.0;6.5) | 20.9 (18.6;23.2) | 39.8 (34.6;45.0) | 46.9 (37.9;55.9) |
| **Self-injury within the last year^1^** | 4.5 (3.9;5.0) | 18.0 (16.5;19.6) | 45.4 (41.7;49.1) | 55.1 (49.9;60.3) | 1.9  (1.5;2.3) | 10.2 (8.4;11.9) | 26.6 (21.8;31.4) | 44.2 (35.3;53.1) |
| **Disordered eating behavior symptoms^2^** | 26.9 (25.7;28.2) | 38.9 (36.9;40.9) | 48.3 (44.4;52.1) | 62.1 (56.8;67.4) | 30.0 (28.5;31.5) | 30.4 (27.8;33.0) | 31.4 (26.4;36.4) | 34.2 (25.2;43.2) |
| **Social anxiety^2^** | 7.9 (7.1;8.6) | 19.6 (17.9;21.2) | 30.0 (26.4;33.5) | 39.3 (33.9;44.8) | 7.5  (6.6;8.4) | 22.4 (20.0;24.9) | 29.7 (24.9;34.5) | 31.5 (22.6;40.5) |
| **Panic anxiety^2^** | 5.9 (5.2;6.5) | 19.8 (18.2;21.5) | 36.5 (32.7;40.2) | 52.9 (47.3;58.4) | 7.8 (6.9;8.8) | 22.1 (19.7;24.6) | 38.2 (32.9;43.5) | 43.3 (33.8;52.7) |
| **Externalizing problems^2^** | 4.3 (3.7;4.9) | 10.5 (9.2;11.8) | 17.1 (14.1;20.2) | 30.3 (25.0;35.5) | 6.5  (5.6;7.3) | 14.6 (12.4;16.7) | 23.8 (18.9;28.6) | 24.2 (16.0;32.5) |
| **Internalizing problems^2^** | 3.2 (2.7;3.8) | 13.5 (12.0;15.0) | 26.8 (23.3;30.3) | 36.0 (30.6;41.5) | 4.1  (3.4;4.8) | 15.7 (13.6;17.9) | 31.6 (26.4;36.7) | 43.5 (34.0;53.0) |
| **Low Quality of life^1^** | 14.1 (13.2;15.1) | 35.5 (33.6;37.5) | 52.3 (48.6;56.0) | 59.6 (54.5;64.7) | 9.5 (8.5;10.5) | 30.1 (27.5;32.7) | 47.4 (42.2;52.6) | 53.7 (44.8;62.7) |
| **Self-assessed health^1^** | 6.3 (5.6;7.0) | 15.7 (14.2;17.3) | 29.5 (26.1;32.9) | 35.9 (30.9;41.0) | 4.4  (3.7;5.1) | 12.3 (10.5;14.2) | 21.6 (17.4;25.9) | 31.8 (23.5;40.2) |
| **Risk behavior** | | | | | | | | |
| **Alcohol dependence^1^** | 8.0 (7.3;8.7) | 12.5 (11.2;13.9) | 11.1 (8.8;13.4) | 18.3 (14.3;22.3) | 12.5 (11.5;13.5) | 14.0 (12.2;15.9) | 17.6 (13.6;21.5) | 23.4 (15.9;30.9) |
| **Smokes daily^1^** | 4.0 (3.4;4.6) | 7.4 (6.3;8.6) | 13.9 (11.1;16.6) | 34.5 (29.4;39.6) | 5.7 (4.9;6.5) | 7.7 (6.2;9.2) | 13.8 (9.9;17.6) | 28.2 (20.0;36.4) |
| **Tried cannabis within the last year^2^** | 12.0 (11.1;12.9) | 23.0 (21.2;24.7) | 28.6 (25.1;32.1) | 41.9 (36.4;47.4) | 23.7 (22.3;25.1) | 33.3 (30.7;36.0) | 34.0 (28.8;39.2) | 42.1 (32.7;51.5) |
| **Tried drugs within the last year^2^** | 2.0 (1.6;2.4) | 5.6 (4.6;6.5) | 10.4 (7.7;13.1) | 17.9 (13.6;22.2) | 6.5  (5.7;7.3) | 11.1 (9.3;12.9) | 15.6 (11.6;19.6) | 28.4 (19.3;37.4) |
| **Sleep deficiency (<7 hours/night)^2^** | 6.8 (6.1;7.6) | 12.2 (10.8;13.7) | 16.7 (13.7;19.8) | 20.0 (15.5;24.5) | 12.3 (11.2;13.4) | 18.6 (16.3;20.9) | 23.6 (18.8;28.4) | 31.6 (22.6;40.7) |
| **Social life factors** | | | | | | | | |
| **Loneliness^1^** | 9.2 (8.4;10.0) | 28.2 (26.4;30.0) | 41.3 (37.6;45.0) | 47.3 (42.0;52.5) | 4.9 (4.2;5.6) | 20.2 (18.0;22.5) | 41.6 (36.4;46.8) | 41.7 (32.9;50.5) |
| **Difficulties making friends^1^** | 19.3 (18.2;20.4) | 33.1 (31.2;35.0) | 42.6 (38.9;46.3) | 43.9 (38.7;49.2) | 14.8 (13.7;15.9) | 29.5 (26.9;32.0) | 40.5 (35.4;45.6) | 44.0 (35.1;53.0) |
| **Low social abilities^2^** | 9.4 (8.6;10.2) | 13.0 (11.6;14.4) | 15.3 (12.5;18.1) | 11.0 (7.6;14.4) | 10.3 (9.3;11.3) | 12.7 (10.9;14.6) | 15.3 (11.5;19.1) | 13.8 (7.4;20.1) |
| ^1^ N=11,274 (girls), N=7908 (boys)  ^2^ N=10,615 (girls), N=7214 (boys) | | | | | | | | |

**Table S6.** Weighted mean scores of characteristics on continuous scales in 18-year-olds according to suicidality, (mutually exclusive categories defined as no suicidality, self-reported suicide ideation, self-reported suicide attempt, and hospital-recorded suicide attempt) presented with 95% confidence intervals.

|  | **Self-reported** | | | | | **Hospital recorded** | |
| --- | --- | --- | --- | --- | --- | --- | --- |
|  | **No suicidality**  **♀ N=16,740**  **♂ N=14,225** | **Vs** | **Suicide ideation**  **♀ N=9763**  **♂ N=5491** | **Vs** | **Suicide attempt**  **♀ N=854**  **♂ N=359** | **Vs** | **Suicide attempt**  **♀ N=366**  **♂ N=54** |
|  | **Mean (CI 95%)** | **P-value** | **Mean (CI 95%)** | **P-value** | **Mean (CI 95%)** | **P-value** | **Mean (CI 95%)** |
| **Girls (27 723 / 26 131)^1,2^** | | | | | | | |
| **Mental health and well-being** | | | | | | | |
| **Depressive symptoms^2,3^ (0-50)** | 10.9 (10.8;11.1) | <0.001 | 19.3  (19.1;19.5) | <0.001 | 25.5  (24.7;26.3) | 0.923 | 25.6  (24.2;27.0) |
| **Mental well-being^1,4^**  **(7-35)** | 24.0 (24.0;24.1) | <0.001 | 22.0  (21.9;22.0) | <0.001 | 20.9  (20.7;21.1) | 0.110 | 20.6  (20.3;21.0) |
| **Social anxiety^2,3^**  **(0-18)** | 6.3  (6.2;6.3) | <0.001 | 8.6  (8.5;8.7) | <0.001 | 9.9  (9.5;10.2) | 0.147 | 10.2  (9.7;10.7) |
| **Panic anxiety^2,3^**  **(0-27)** | 2.4  (2.4;2.5) | <0.001 | 5.6  (5.5;5.7) | <0.001 | 9.2  (8.7;9.7) | 0.428 | 9.4  (8.6;10.1) |
| **Externalizing problems^2,3^**  **(0-20)** | 4.9  (4.9;5.0) | <0.001 | 6.6  (6.5;6.6) | <0.001 | 8.4  (8.2;8.7) | 0.148 | 8.2  (7.8;8.5) |
| **Internalizing problems^2,3^  (0-20)** | 5.2  (5.2;5.3) | <0.001 | 8.0  (7.9;8.0) | <0.001 | 9.9  (9.6;10.1) | 0.398 | 9.7  (9.4;10.1) |
| **Quality of life^1,4^ (0-10)** | 7.2  (7.2;7.3) | <0.001 | 6.0  (5.9;6.0) | <0.001 | 5.2  (5.0;5.3) | 0.001 | 4.9  (4.6;5.1) |
| **Risk behavior** | | | | | | | |
| **Alcohol dependence^1,3^ (0-40)** | 7.9  (7.8;8.0) | <0.001 | 8.4  (8.3;8.5) | <0.001 | 9.2  (8.8;9.6) | 0.105 | 8.8  (8.1;9.4) |
| **Social life factors** | | | | | | | |
| **Low social abilities^2,3^ (0-10)** | 8.3  (8.3;8.4) | <0.001 | 8.2  (8.1;8.2) | 0.124 | 8.2  (8.1;8.3) | 0.656 | 8.3  (8.1;8.4) |
| **Boys (N=20 129 / 18 448)^1,2^** | | | | | | | |
| **Mental health and well-being** | | | | | | | |
| **Depressive symptoms^2,3^ (0-50)** | 7.2  (7.1;7.3) | <0.001 | 14.6  (14.3;14.9) | <0.001 | 20.5  (19.1;21.8) | 0.004 | 17.5  (14.1;20.8) |
| **Mental well-being^1,4^**  **(7-35)** | 24.9 (24.8;24.9) | <0.001 | 22.5  (22.5;22.6) | <0.001 | 21.5  (21.1;21.9) | 0.535 | 21.7  (20.8;22.6) |
| **Social anxiety^2,3^**  **(0-18)** | 3.7  (3.7;3.8) | <0.001 | 5.8  (5.7;5.9) | <0.001 | 6.6  (6.2;7.1) | 0.021 | 5.6  (4.2;7.0) |
| **Panic anxiety^2,3^**  **(0-27)** | 0.9  (0.9;0.9) | <0.001 | 2.5  (2.4;2.6) | <0.001 | 4.2  (3.7;4.8) | 0.367 | 3.9  (2.5;5.3) |
| **Externalizing problems^2,3^**  **(0-20)** | 5.0  (4.9;5.0) | <0.001 | 6.2  (6.1;6.3) | <0.001 | 7.4  (7.0;7.8) | 0.862 | 7.5  (6.4;8.6) |
| **Internalizing problems^2,3^  (0-20)** | 3.3  (3.3;3.4) | <0.001 | 5.7  (5.6;5.7) | <0.001 | 7.3  (7.0;7.7) | 0.020 | 6.4  (5.3;7.6) |
| **Quality of life^1,4^ (0-10)** | 7.7  (7.7;7.7) | <0.001 | 6.2  (6.1;6.3) | <0.001 | 5.3  (5.1;5.6) | 0.017 | 5.9  (5.2;6.5) |
| **Risk behavior** | | | | | | | |
| **Alcohol dependence^1,3^ (0-40)** | 8.6  (8.5;8.7) | 0.477 | 8.7  (8.5;8.8) | 0.054 | 9.2  (8.4;9.9) | 0.001 | 11.4 (9.7;13.1) |
| **Social life factors** | | | | | | | |
| **Low social abilities^2,3^ (0-10)** | 7.7  (7.7;7.8) | <0.001 | 7.6  (7.6;7.7) | 0.998 | 7.6  (7.4;7.8) | 0.470 | 7.8  (7.3;8.3) |
| ^1^ N=27,723 (girls), N=20,129 (boys), ^2^ N=26,131 (girls), N=18,448 (boys)  ^3^ Lower score is better, ^4^ Higher score is better | | | | | | | |

**Table S7.** Weighted mean scores of characteristics on continuous scales in 18-year-olds according to suicidality, (mutually exclusive categories defined as no suicidality, self-reported suicide ideation, self-reported suicide plans, and self-reported or hospital-recorded suicide attempt) presented with 95% confidence intervals.

|  | **Self-reported** | | | | | **Hospital recorded/self-reported** | |
| --- | --- | --- | --- | --- | --- | --- | --- |
|  | **No suicidality ♀ N=6816**  **♂ N=5436** | **Vs** | **Suicide ideation ♀ N=3100**  **♂ N=1818** | **Vs** | **Suicide plans ♀ N=899**  **♂ N=439** | **Vs** | **Suicide attempt ♀ N=459**  **♂ N=161** |
|  | **Mean (CI 95%)** | **P-value** | **Mean (CI 95%)** | **P-value** | **Mean (CI 95%)** | **P-value** | **Mean (CI 95%)** |
| **Girls (11 274 / 10 615)^1,2^** | | | | | | | |
| **Mental health and well-being** | | | | | | | |
| **Depressive symptoms^2,3^ (0-50)** | 11.5 (11.3;11.7) | <0.001 | 18.7  (18.4;19.1) | <0.001 | 23.7  (22.9;24.5) | <0.001 | 27.3  (26.1;28.4) |
| **Mental well-being^1,4^**  **(7-35)** | 24.1 (24.0;24.1) | <0.001 | 22.2  (22.1;22.3) | <0.001 | 21.1  (20.9;21.3) | <0.001 | 20.6  (20.3;20.9) |
| **Social anxiety^2,3^**  **(0-18)** | 6.5  (6.4;6.6) | <0.001 | 8.6  (8.4;8.7) | <0.001 | 10.0  (9.7;10.2) | <0.001 | 10.7  (10.3;11.1) |
| **Panic anxiety^2,3^**  **(0-27)** | 2.6  (2.6;2.7) | <0.001 | 5.7  (5.5;5.8) | <0.001 | 8.1  (7.7;8.6) | <0.001 | 10.8  (10.1;11.5) |
| **Externalizing problems^2,3^**  **(0-20)** | 5.0  (4.9;5.1) | <0.001 | 6.4  (6.3;6.5) | <0.001 | 7.3  (7.1;7.6) | <0.001 | 8.7  (8.4;9.0) |
| **Internalizing problems^2,3^  (0-20)** | 5.2  (5.2;5.3) | <0.001 | 7.8  (7.7;7.9) | <0.001 | 9.2  (9.0;9.4) | <0.001 | 10.3  (9.9;10.6) |
| **Quality of life^1,4^ (0-10)** | 7.2  (7.1;7.2) | <0.001 | 6.0  (6.0;6.1) | <0.001 | 5.3  (5.1;5.4) | 0.002 | 5.0  (4.8;5.2) |
| **Risk behavior** | | | | | | | |
| **Alcohol dependence^1,3^ (0-40)** | 7.9  (7.8;8.0) | <0.001 | 8.5  (8.3;8.6) | 0.058 | 8.1  (7.8;8.5) | <0.001 | 9.1  (8.6;9.7) |
| **Social life factors** | | | | | | | |
| **Social abilities^2,3^ (0-10)** | 8.4  (8.3;8.4) | <0.001 | 8.2  (8.1;8.2) | 0.672 | 8.1  (8.0;8.3) | 0.009 | 8.4  (8.2;8.5) |
| **Boys (N=7908 / 7214)^1,2^** | | | | | | | |
| **Mental health and well-being** | | | | | | | |
| **Depressive symptoms^2,3^ (0-50)** | 7.5  (7.3;7.6) | <0.001 | 13.8  (13.4;14.2) | <0.001 | 18.9  (17.9;19.9) | <0.001 | 21.8  (19.8;23.9) |
| **Mental well-being^1,4^**  **(7-35)** | 24.9 (24.8;25.0) | <0.001 | 22.9  (22.7;23.0) | <0.001 | 21.5  (21.2;21.8) | 0.392 | 21.3  (20.7;21.9) |
| **Social anxiety^2,3^**  **(0-18)** | 3.8  (3.7;3.9) | <0.001 | 5.9  (5.7;6.1) | 0.006 | 6.3  (5.9;6.7) | 0.066 | 6.9  (6.1;7.6) |
| **Panic anxiety^2,3^**  **(0-27)** | 1.0  (0.9;1.0) | <0.001 | 2.3  (2.1;2.4) | <0.001 | 3.6  (3.2;4.0) | <0.001 | 5.0  (4.1;5.9) |
| **Externalizing problems^2,3^**  **(0-20)** | 5.0  (4.9;5.0) | <0.001 | 6.0  (5.9;6.2) | <0.001 | 6.8  (6.4;7.1) | <0.001 | 7.8  (7.2;8.4) |
| **Internalizing problems^2,3^  (0-20)** | 3.3  (3.3;3.4) | <0.001 | 5.4  (5.2;5.5) | <0.001 | 6.9  (6.6;7.2) | <0.001 | 7.7  (7.1;8.3) |
| **Quality of life^1,4^ (0-10)** | 7.6  (7.6;7.7) | <0.001 | 6.3  (6.2;6.4) | <0.001 | 5.4  (5.2;5.6) | 0.031 | 5.1  (4.7;5.5) |
| **Risk behavior** | | | | | | | |
| **Alcohol dependence^1,3^ (0-40)** | 8.5  (8.3;8.6) | 0.325 | 8.6  (8.4;8.9) | 0.694 | 8.7  (8.1;9.3) | 0.025 | 9.7  (8.6;10.8) |
| **Social life factors** | | | | | | | |
| **Social abilities^2,3^ (0-10)** | 7.8  (7.8;7.9) | <0.001 | 7.6  (7.6;7.7) | 0.201 | 7.5  (7.3;7.7) | 0.736 | 7.6  (7.2;7.9) |
| ^1^ N=11,274 (girls), N=7908 (boys), ^2^ N=10,615 (girls), N=7214 (boys)  ^3^ Lower score is better, ^4^ Higher score is better | | | | | | | |

**Table S8.** Weighted proportion of characteristics in 18-year-olds relying ‘no’ and ‘do not know’ to suicidality in DNBC-18 (previously presented as one group), presented with 95% confidence intervals.

|  | **Girls (16,740 / 15,787)^1,2^** | | **Boys (N=14,225 / 12,971)^1,2^** | |
| --- | --- | --- | --- | --- |
|  | **No suicidality**  **N=15,362**  **% (CI 95%)** | **Do not know**  **N=1378**  **% (CI 95%)** | **No suicidality**  **N=13,417**  **% (CI 95%)** | **Do not  know**  **N=808**  **% (CI 95%)** |
| **Mental health and well-being** | | | | |
| **Depressive symptoms^2^** | 11.9 (11.3;12.5) | 30.5 (27.7;33.4) | 4.2 (3.7;4.6) | 14.2 (11.5;17.0) |
| **Poor mental well-being^1^** | 7.7 (7.2;8.2) | 21.9 (19.4;24.5) | 5.1 (4.7;5.6) | 20.0 (16.8;23.1) |
| **Self-injury within the last year^1^** | 4.3 (3.9;4.6) | 9.0 (7.2;10.7) | 2.1 (1.8;2.4) | 4.3  (2.6;5.9) |
| **Disordered eating behavior symptoms^2^** | 26.2 (25.4;27.0) | 35.2 (32.5;38.2) | 28.7 (27.8;29.6) | 26.0 (22.4;29.5) |
| **Social anxiety^2^** | 6.3 (5.9;6.8) | 13.3 (11.2;15.4) | 6.8 (6.3;7.4) | 15.4 (12.4;18.5) |
| **Panic anxiety^2^** | 4.2 (3.9;4.6) | 10.0 (8.1;11.9) | 6.2 (5.7;6.8) | 20.3 (16.8;23.8) |
| **Externalizing problems^2^** | 3.9 (3.5;4.3) | 5.7 (4.3;7.2) | 6.6 (6.1;7.2) | 11.9 (9.2;14.6) |
| **Internalizing problems^2^** | 2.7 (2.4;3.0) | 7.3 (5.6;9.0) | 3.4 (3.0;3.8) | 12.0 (9.1;14.8)) |
| **Low quality of life^1^** | 11.6 (11.1;12.2) | 28.5 (25.7;31.2) | 7.5 (7.0;8.0) | 24.9 (21.5;28.2) |
| **Self-assessed health^1^** | 6.2 (5.8;6.7) | 10.6 (8.7;12.5) | 4.1 (3.7;4.5) | 10.0 (7.6;12.4) |
| **Risk behavior** | | | | |
| **Alcohol dependence^1^** | 7.3  (6.9;7.8) | 8.8 (7.1;10.4) | 12.4 (11.8;13.1) | 13.5 (10.8;16.2) |
| **Smokes daily^1^** | 5.7 (5.3;6.2) | 5.9 (4.3;7.5) | 8.6 (8.0;9.2) | 9.4  (6.8;12.1) |
| **Tried cannabis within the last year^2^** | 13.2 (12.6;13.8) | 16.5 (14.2;18.7) | 25.8 (25.0;26.7) | 27.2 (23.6;30.9) |
| **Tried drugs within the last year^2^** | 2.4 (2.1;2.7) | 2.5  (1.5;3.5) | 8.1 (7.6;8.7) | 8.6  (6.2;11.0) |
| **Sleep deficiency (<7 hours/night)^2^** | 5.9 (5.4;6.3) | 9.8 (7.9;11.8) | 12.3 (11.6;13.0) | 16.1 (12.9;19.3) |
| **Social life factors** | | | | |
| **Loneliness^1^** | 7.6 (7.1;8.0) | 17.1 (14.9;19.4) | 4.0 (3.7;4.4) | 14.1 (11.4;16.9) |
| **Difficulties making friends^1^** | 18.3 (17.6;19.0) | 26.6 (24.0;29;2) | 13.7 (13.0;14.4) | 22.5 (19.3;25.7) |
| **Low social abilities^2^** | 10.1 (9.6;10.7) | 13.9 (11.7;16.0) | 10.9 (10.3;11.6) | 17.0 (13.7;20.2) |
| ^1^ N=16,740 (girls), N=14,225 (boys) ^2^ N=15,787 (girls), N=12,971 (boys) | | | | |

**Figure S1.** Flowchart of the study population.


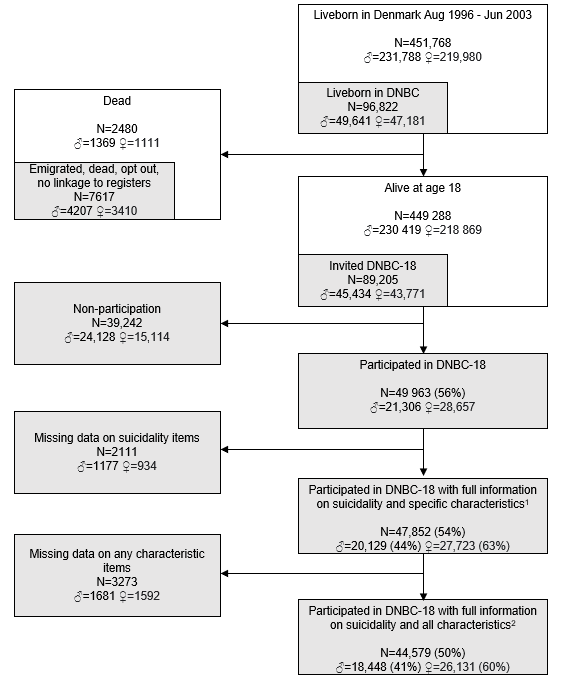


^1^Mental well-being, self-injury within the past year, internalizing problems, externalizing problems, quality of life, self-assessed health, alcohol dependence, daily smoking, sleep deficiency (<7 hours/night), loneliness, difficulties making friends, and low social abilities.

^2^Depressive symptoms, mental well-being, self-injury within the past year, disordered eating behavior symptoms, social anxiety symptoms, panic anxiety symptoms, internalizing problems, externalizing problems, quality of life, self-assessed health, alcohol dependence, daily smoking, tried cannabis within the last year, tried drugs within the last year, sleep deficiency (<7 hours/night), loneliness, difficulties making friends, and low social abilities.

**Figure S2.** Weighted proportion of poor mental health and well-being in 18-year-olds according to recency of suicidality (mutually exclusive categories of no suicidality, past suicidality (more than one year ago), and current suicidality (within the last year) with suicidality defined as either self-reported suicide ideation, self-reported suicide attempt, or hospital-recorded suicide attempt. Presented with 95% confidence intervals and p-values comparing proportions between groups.

a) Girls in the central study population (N=27,723)

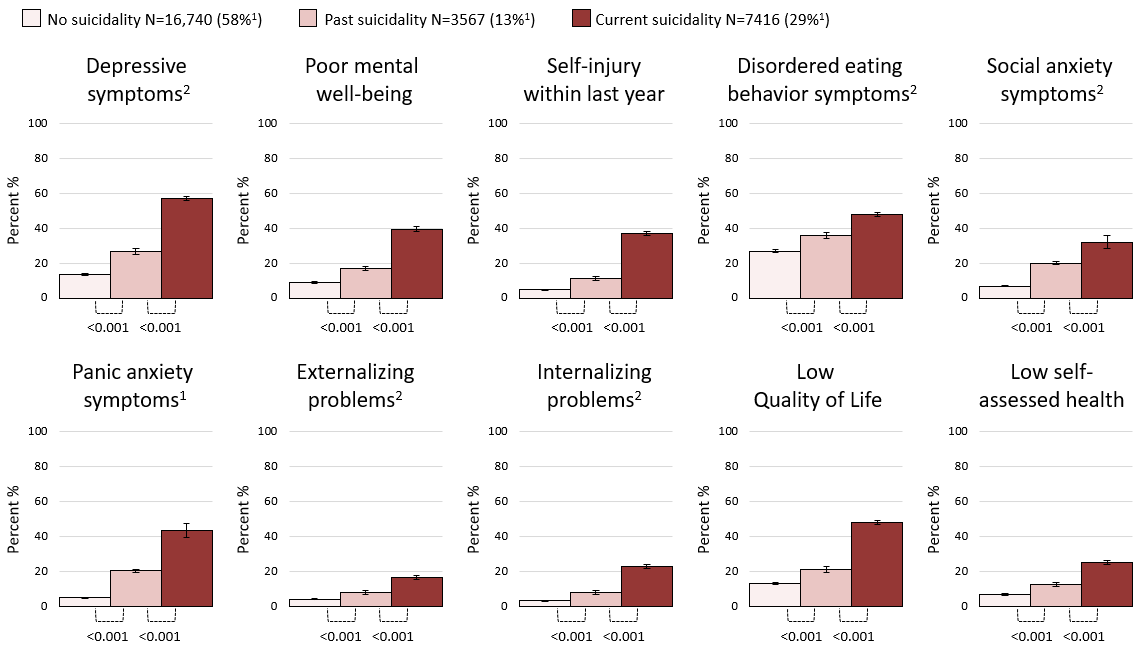


a) Boys in the central study population (N=20,129)


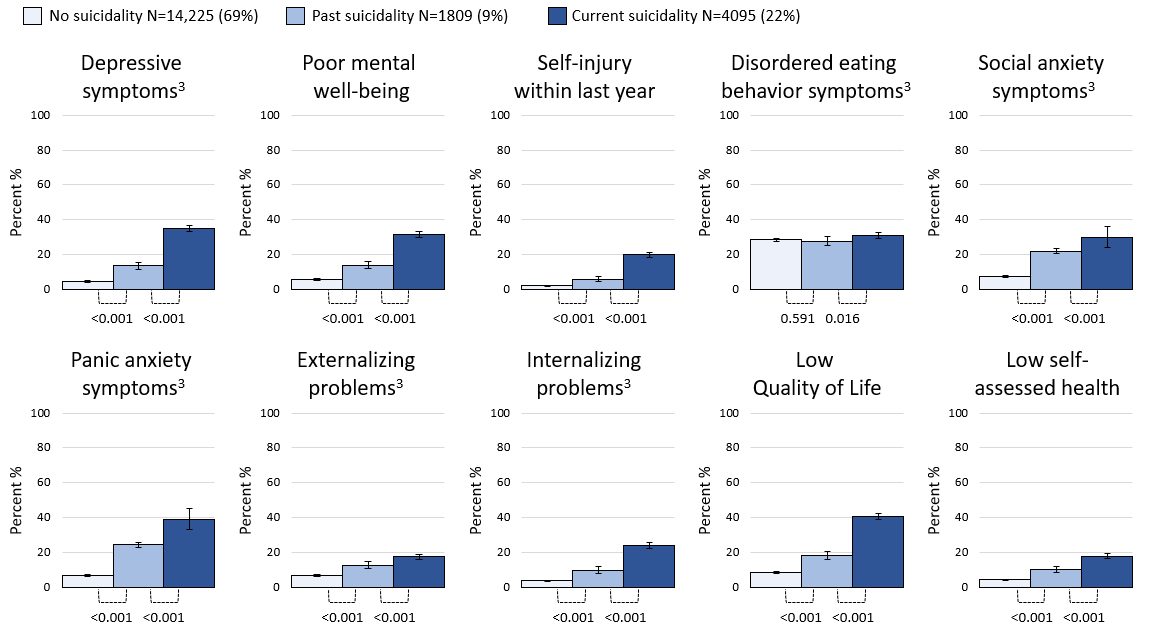
^1^Fewer observations due to missing values on characteristics, N=26,131, ^2^Fewer observations due to missing values on characteristics, N=18,448
